# Supplementary material for: Peculiar transient behaviors of organic electrochemical transistors governed by ion injection directionality
Source: Nat Commun. 2023 Nov 28;14:7577. doi: 10.1038/s41467-023-42840-z (PMC10684893; doi:10.1038/s41467-023-42840-z)
Supplement: Supplementary file 1 — Supplementary Information [file 41467_2023_42840_MOESM1_ESM.pdf]

Supplementary Information for

Peculiar Transient Behaviors of Organic Electrochemical Transistors Governed by Ion Injection Directionality

Ji Hwan Kim,[1†] Roman Halaksa,[2†] Il-Young Jo,[1] Hyungju Ahn,[3] Peter A. Finn,[2] Inho Lee,[4] Sungjun Park,[4] Christian B. Nielsen,[2]* and Myung-Han Yoon[1]*

[1]School of Materials Science and Engineering, Gwangju Institute of Science and Technology (GIST), Gwangju 61005, Republic of Korea
[2]Department of Chemistry, Queen Mary University of London, London E14NS, United Kingdom
[3]Pohang Accelerator Laboratory, Pohang 37673, Republic of Korea
[4]Department of Electrical and Computer Engineering, Ajou University, Suwon 16499, Republic of Korea

[†]These authors contributed equally to this research.

*Corresponding author. Email: c.b.nielsen@qmul.ac.uk (C.B.N.) and mhyoon@gist.ac.kr (M.-H.Y.)

Supplementary Text

<u>Synthetic Procedures</u>

The synthesis of the target polymers started with alkylation of molecule **1** with molecule **2** in a yield of 46 %. Subsequently, molecule **5** was synthesized by a direct arylation reaction of molecules **3** and **4** in a yield of 77 %. This molecule was reacted with TBAF to give molecule **6** in 89% yield. The last step was the direct arylation reaction of molecule **6** with 1,4-dibromobenzene and 5,5′-Dibromo-2,2′-bithiophene to give the target polymers DTP-P and DTP-2T, respectively. Both target polymers were precipitated into hexane and washed with hexane, methanol, and acetone via Soxhlet extraction. Finally, chloroform was used to wash target polymers off the thimble, and these were recovered after solvent evaporation.

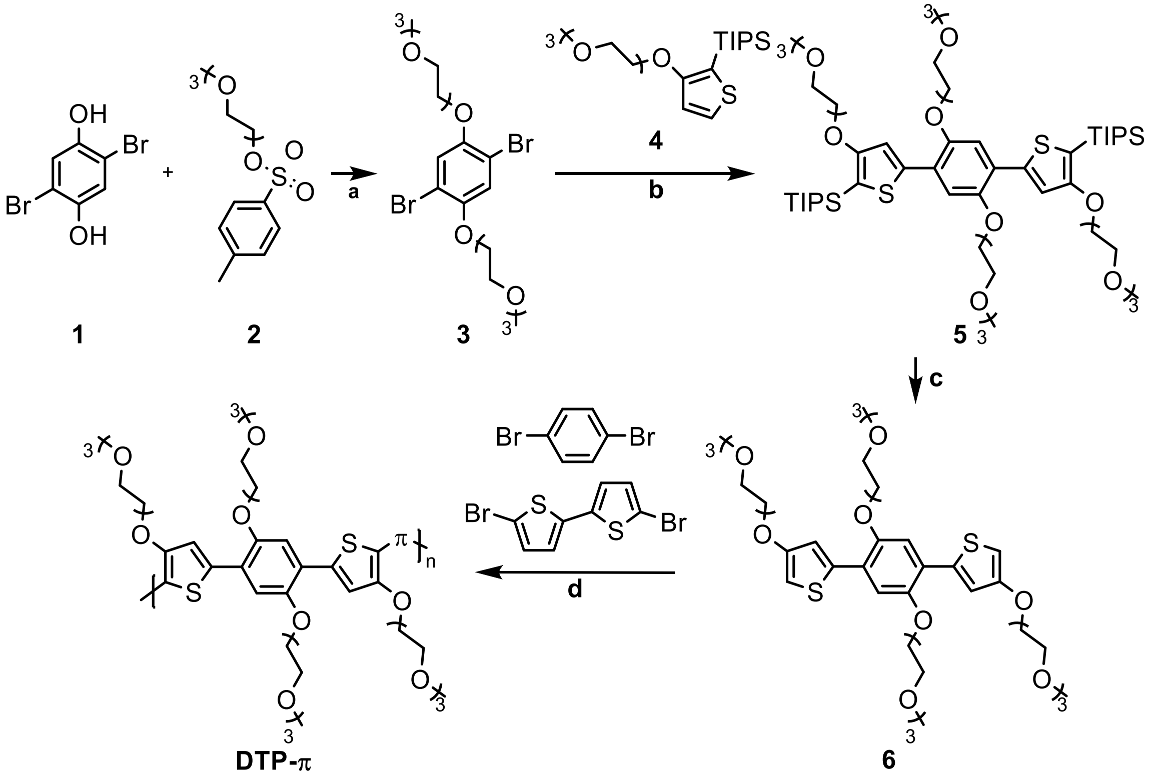

Synthesis of target polymers (DTP-P, DTP-2T), a) K_2CO_3, DMF, 70 °C, b) $Pd(OAc)_2$, tri(2-methoxyphenyl)phosphine, Cs_2CO_3, pivalic acid, toluene, 120 °C, c) TBAF, THF, RT, d) $Pd(OAc)_2$, tri(2-methoxyphenyl)phosphine, Cs_2CO_3, pivalic acid, chlorobenzene, 120 °C.

Synthesis

2,5-Dibromohydroquinone (1) was prepared using previously reported procedure with yield 48 % (ref. 55).

2-(2-(2-methoxyethoxy)ethoxy)ethyl benzenesulfonate (2) was prepared using previously reported procedure with yield 93 % (ref. 56).

triisopropyl(3-(2-(2-(2-methoxyethoxy)ethoxy)ethoxy)thiophen-2-yl)silane (4) was prepared using previously reported procedure with yield 71 % (ref. 30).

1,4-dibromo-2,5-bis[2-[2-(2-methoxyethoxy)ethoxy]ethoxy]benzene (3)

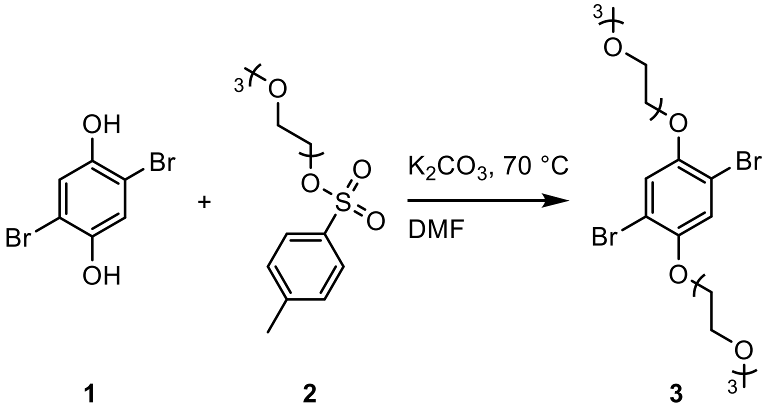

2,5-Dibromohydroquinone (3.18 g, 19.0 mmol) and 2-(2-(2-methoxyethoxy)ethoxy)ethyl 4-methylbenzenesulfonate (5.78 g, 41.8 mmol) were dissolved in anhydrous DMF (150 ml). Then potassium carbonate (5.78 g, 41.8 mmol) was added at RT and temperature was raised to 70 °C and reaction mixture was stirred for 30 hours. Reaction mixture was poured into mixture of water and saturated aqueous ammonium chloride solution (300 ml) (1/1) and extracted with diethylether (4x100 ml). Combined organic extracts were washed with water (3x100 ml), brine (100 ml), dried ($MgSO_4$) and evaporated. Obtained crude product was dissolved in mixture of hexane and ethyl-acetate (1/1) and filtered through silica pad and evaporated. Crude product was dissolved in ethanol (40 ml) and solution of potassium hydroxide (3.54 g. 63.1 mmol) dissolved in water (40 ml) was added and reaction mixture was stirred at 70 °C overnight. Reaction mixture was poured into water (100 ml) and extracted with diethylether (3x100 ml). Combined organic extracts were washed with water (3x100), brine (2x100 ml), dried ($MgSO_4$) and evaporated to obtain product as a light orange oil (4.87 g, 46 %). ^{1}H NMR (400 MHz, CDCl3) δ 7.14 (s, 2H), 4.14 – 4.10 (m, 4H), 3.88 – 3.85 (m, 4H), 3.78 – 3.75 (m, 4H), 3.70 – 3.63 (m, 8H), 3.57 – 3.52 (m, 4H), 3.37 (s, 6H).

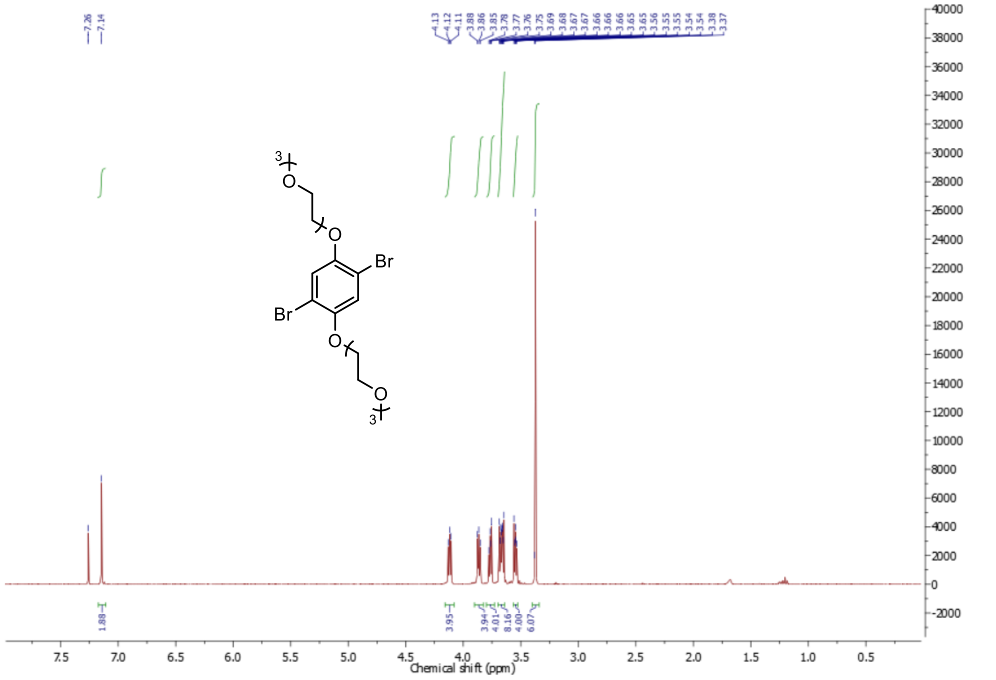

Chemical shift (ppm)
Br
Br

((2,5-bis(2-(2-(2-methoxyethoxy)ethoxy)ethoxy)-1,4-phenylene)bis(3-(2-(2-(2-methoxyethoxy)ethoxy)ethoxy)thiophene-5,2-diyl))bis(triisopropylsilane) (5)

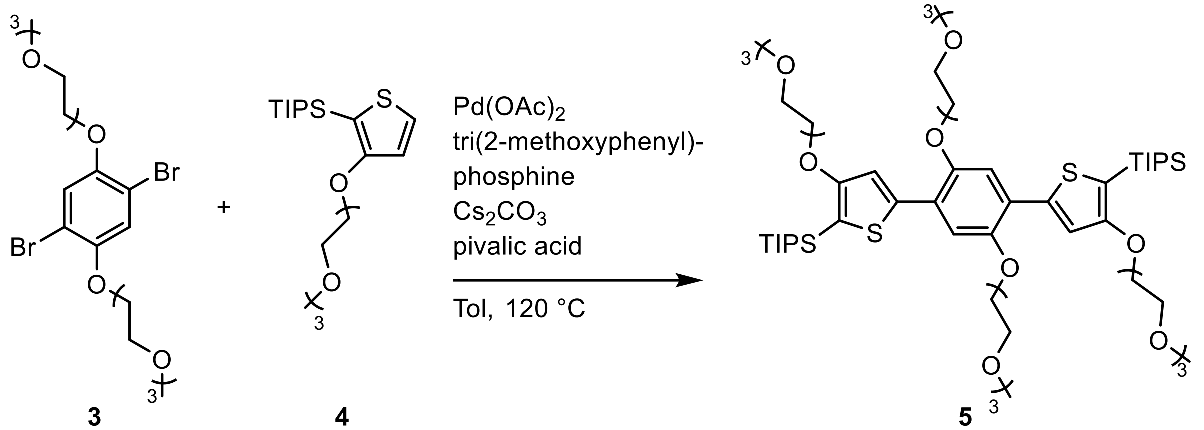

3 4 5

Palladium acetate (25.6 mg, 0.11 mmol), tris(2-methoxyphenyl)phosphine (80.5 mg, 0.22 mmol), caesium carbonate (2.79 g, 8.56 mmol), pivalic acid (0.58 g, 5.71 mmol) were mixed in sealed vial and purged with nitrogen 15 min. Anhydrous toluene (23 ml) was added, followed by compound **4** (2.30 g, 7.71 mmol), compound **3** (1.44 g, 2.57 mmol) and reaction mixture was bubbled with nitrogen 15 min. Temperature was raised to 120 °C and reaction mixture was stirred 60 hours. After cooling down reaction mixture was diluted with chloroform (50 ml) and filtered through celite pad and evaporated. Crude product was purified by silica column chromatography using mixture of toluene and acetone (7/3) as an eluent. Title compound was recovered as a light green oil (2.39 g, 77 %). ^{1}H NMR (400 MHz, CDCl3) δ 7.46 (s, 2H), 7.19 (s, 2H), 4.24 (t, J = 5.0 Hz, 4H), 4.16 (t, J = 5.2 Hz, 4H), 3.94 (t, J = 5.0 Hz, 4H), 3.79 (t, J = 5.1 Hz, 4H), 3.74 – 3.58 (m, 24H), 3.55 – 3.47 (m, 8H), 3.38 – 3.31 (m, 12H), 1.47 – 1.35 (m, 6H), 1.10 (d, J = 7.5 Hz, 36H). ^{13}C NMR (101 MHz, CDCl3) δ 163.20, 149.45, 142.86, 123.26, 115.43, 112.69, 109.69, 71.99, 71.96, 70.87, 70.76, 70.74, 70.64, 70.61, 70.10, 69.93, 69.85, 68.98, 59.06, 59.03, 18.91, 12.15. HRMS Calculated for [C60H106O16S2Si2-H^{+}] 1203.6539, found 1203.6539.

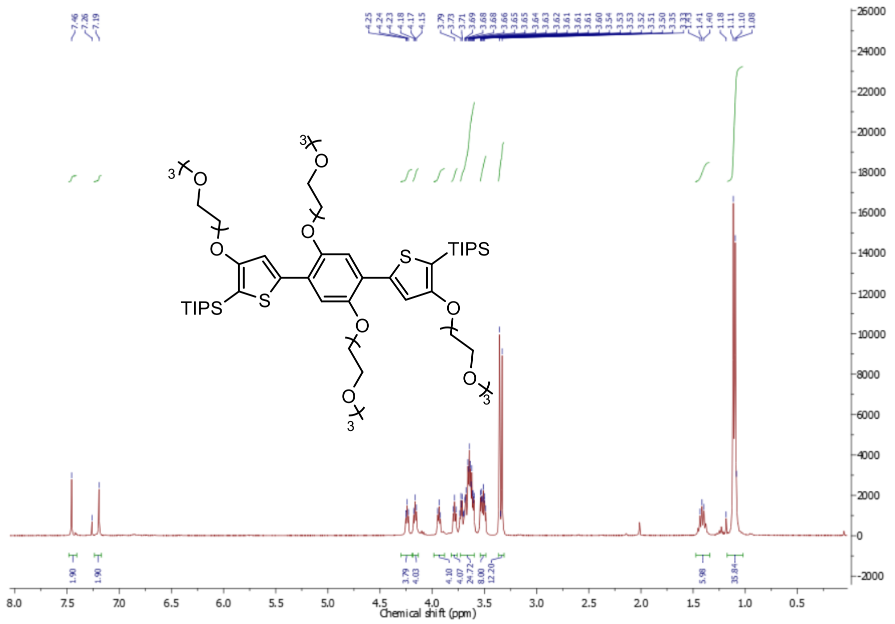
TIPS
S
O
3
Chemical shift (ppm)

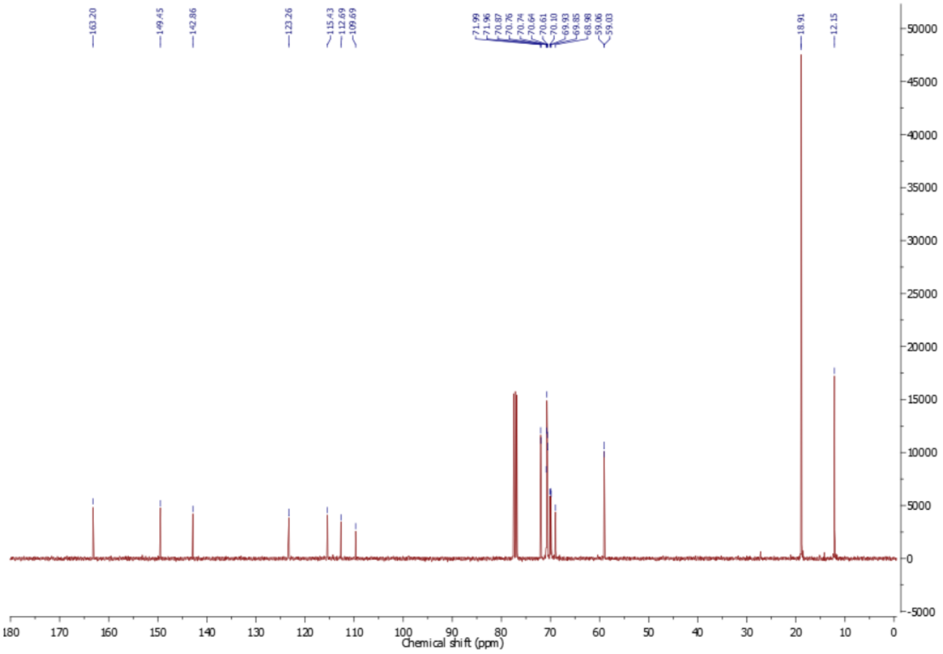
Chemical shift (ppm)

5,5'-(2,5-bis(2-(2-(2-methoxyethoxy)ethoxy)ethoxy)-1,4-phenylene)bis(3-(2-(2-(2-methoxyethoxy)ethoxy)ethoxy)thiophene) (6)

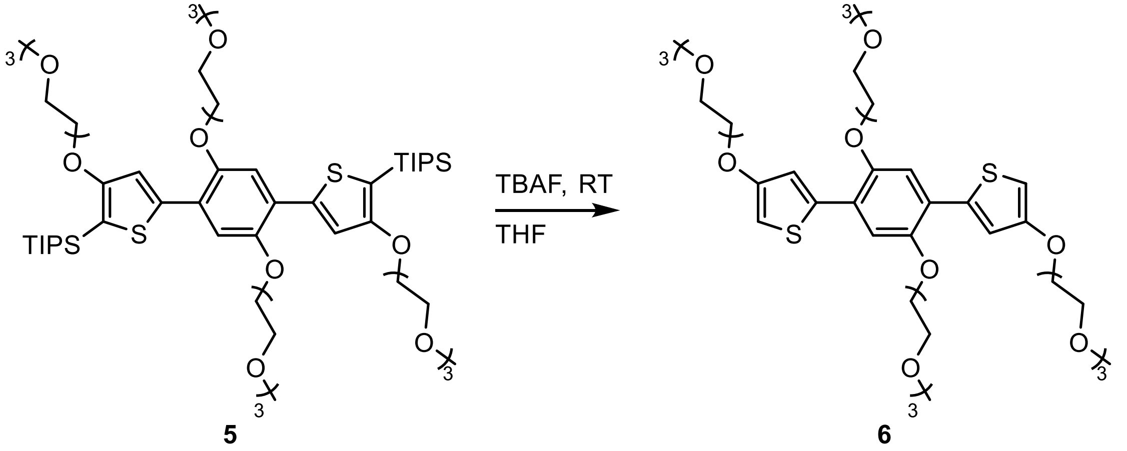

Compound **5** (1.09 g, 0.91 mmol) was dissolved in anhydrous THF (22 ml). Tetrabutylammonium fluoride solution 1.0 M in THF (2.3 ml, 2.26 mmol) was added at room temperature and reaction mixture was stirred at room temperature 2 hours. Then one spatula of ammonium chloride was added and suspension was filtered and evaporated. Crude product was purified by silica column chromatography using mixture of toluene and acetone (1/1) as an eluent. Title compound was recovered as a light brown oil (0.72 g, 89 %). ^{1}H NMR (400 MHz, CDCl3) δ 7.20 (d, J = 1.7 Hz, 2H), 7.14 (s, 2H), 6.22 (d, J = 1.7 Hz, 2H), 4.18 – 4.15 (m, 4H), 4.10 – 4.06 (m, 4H), 3.89 – 3.85 (m, 4H), 3.82 – 3.77 (m, 4H), 3.71 – 3.67 (m, 8H), 3.65 – 3.58 (m, 16H), 3.51 – 3.45 (m, 8H), 3.32 (s, 6H), 3.30 (s, 6H). ^{13}C NMR (101 MHz, CDCl3) δ 156.81, 149.44, 137.42, 123.11, 117.79, 112.81, 98.22, 71.82, 70.75, 70.68, 70.58, 70.55, 70.45, 70.45, 69.65, 69.60, 69.22, 68.98, 58.90, 58.88. HRMS Calculated for [C42H66O16S2-H^{+}] 891.3871, found 891.3870.

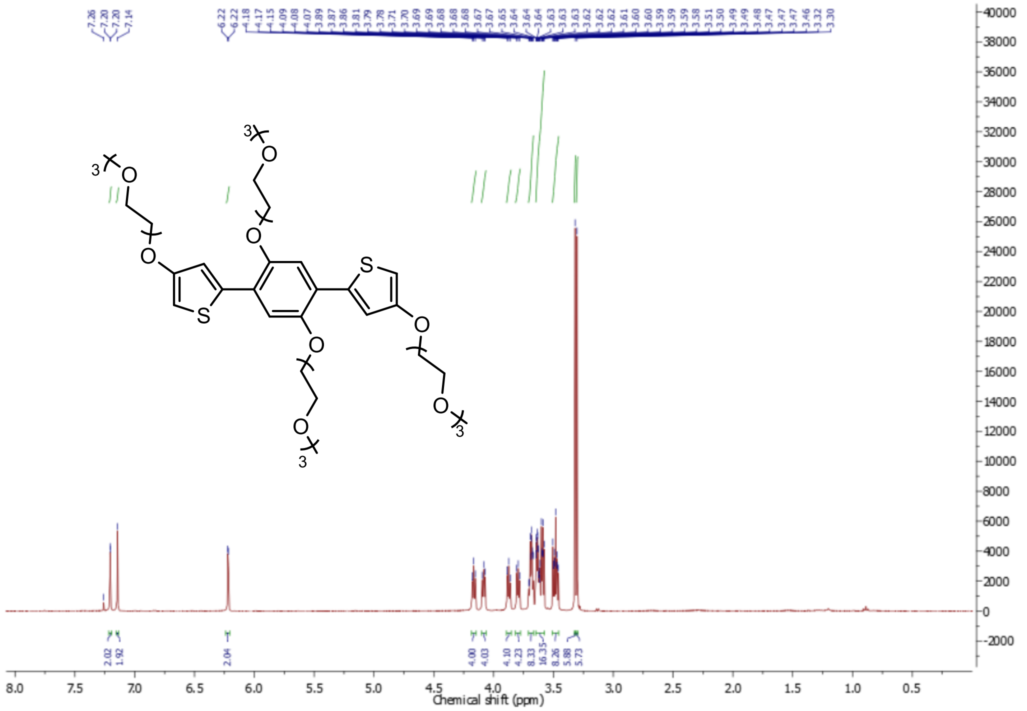
Chemical shift (ppm)

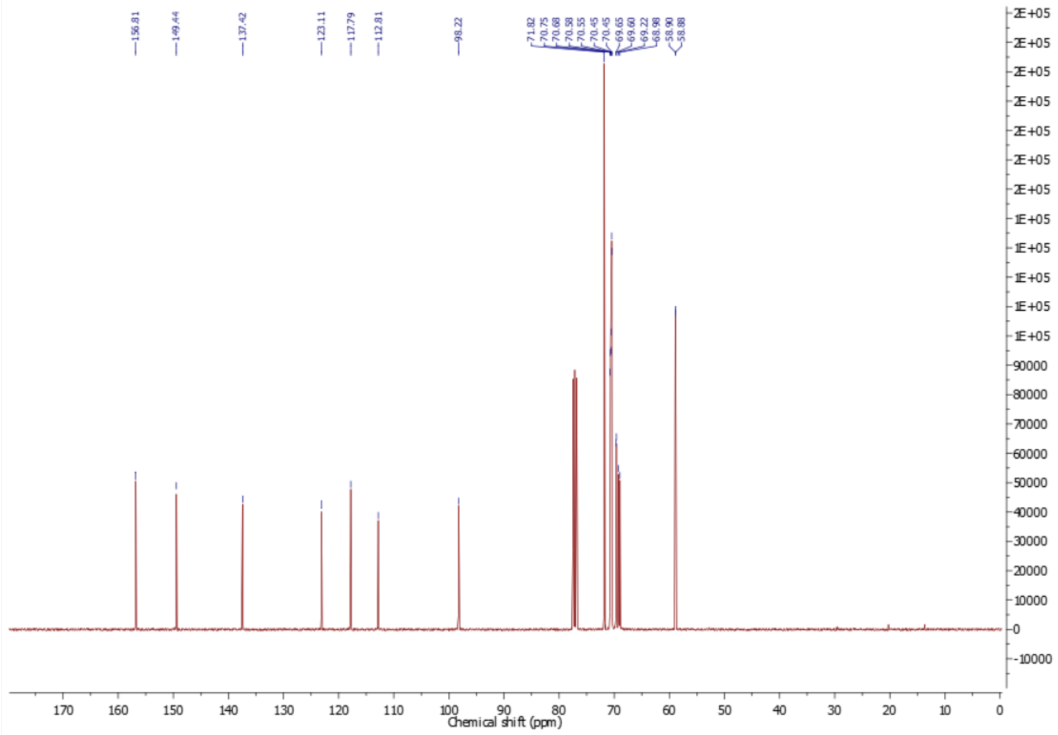
156.81
149.44
137.42
123.11
117.79
112.81
98.22
71.82
70.75
70.68
70.58
70.55
70.45
70.45
69.65
69.60
69.22
68.98
58.90
58.88
Chemical shift (ppm)

General polymerization method

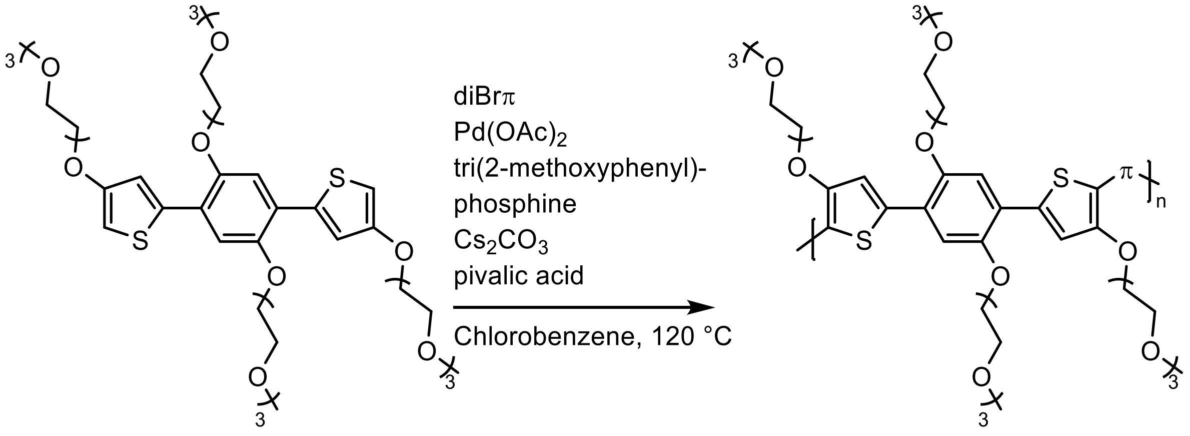

6 **DTP-π**

Palladium acetate (2.0 mg, 0.01 mmol), tris(2-methoxyphenyl)phosphine (6.3 mg, 0.02 mmol), caesium carbonate (0.22 g, 0.67 mmol), pivalic acid (22.9 mg, 0.22 mmol) were mixed in sealed vial and purged with nitrogen 30 min. Anhydrous chlorobenzene (1.5 ml) was added, followed by compound **6** (0.20 g, 0.22 mmol), diBrπ (0.22 mmol) and reaction mixture was bubbled with nitrogen 15 min. Temperature was raised to 120 °C and reaction mixture was stirred 3 days. After cooling down reaction mixture was diluted with chloroform and precipitated into well stirred hexane. Precipitate was transferred into Soxhlet thimble and extracted with hexane (16 h), acetone (16 h) and methanol (4 h). Purified polymer was recovered by extraction with chloroform and evaporation.

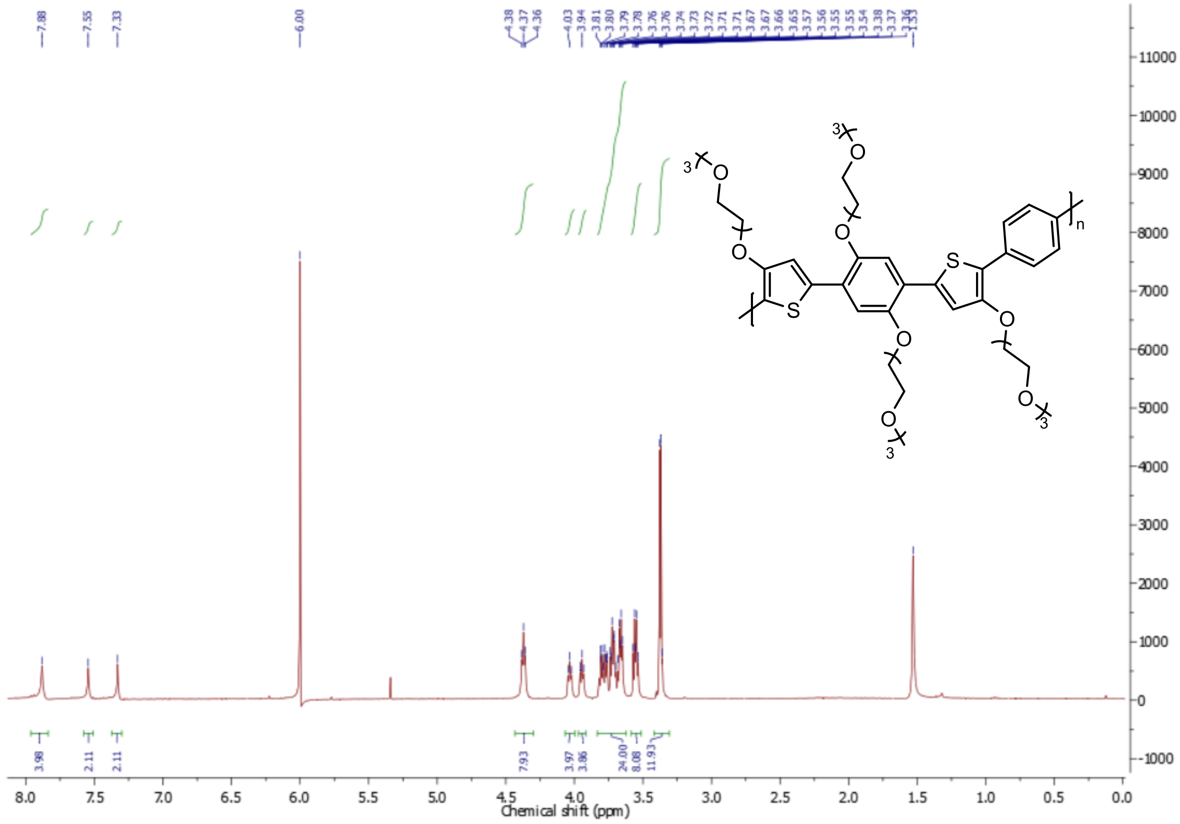
Chemical shift (ppm)

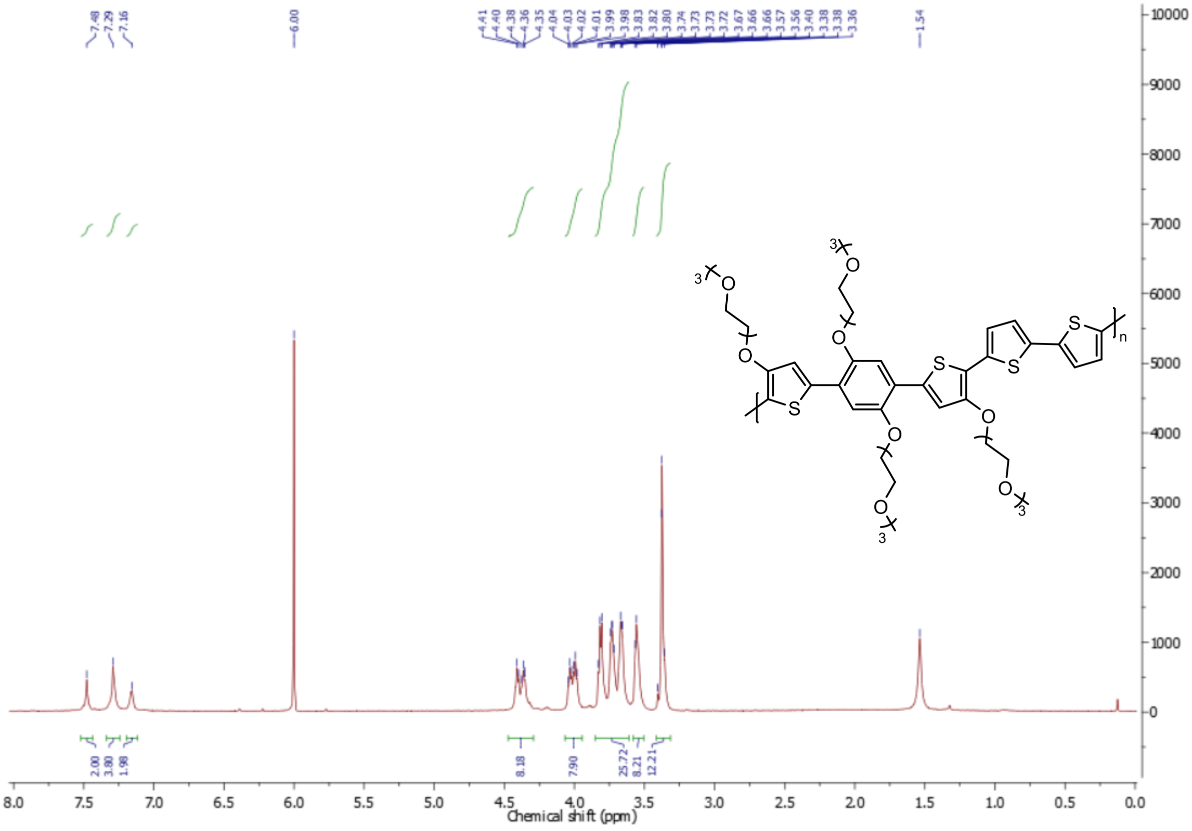
Chemical shift (ppm)

Poly(5-(2,5-bis(2-(2-(2-methoxyethoxy)ethoxy)ethoxy)-4-(4-(2-(2-(2-methoxyethoxy)ethoxy)ethoxy)thiophen-2-yl)phenyl)-3-(2-(2-(2-methoxyethoxy)ethoxy)ethoxy)-2-phenylthiophene) (DTP-P)
1,4-dibromobenzene (52.9 mg, 0.22 mmol), isolated as dark red solid (83 mg, 38 %)

Poly(5-(2,5-bis(2-(2-(2-methoxyethoxy)ethoxy)ethoxy)-4-(4-(2-(2-(2-methoxyethoxy)ethoxy)ethoxy)thiophen-2-yl)phenyl)-3-(2-(2-(2-methoxyethoxy)ethoxy)ethoxy)-2,2':5',2''-terthiophene) (DTP-2T)
5,5′-Dibromo-2,2′-bithiophene (72.7 mg, 0.22 mmol), isolated as dark purple solid (155 mg, 66 %)

Gel permeation chromatography

Molecular weights of DTP-P and DTP-2T are shown in below.

Polymer	M_n [g·mol^{-1}]	M_W [g·mol^{-1}]	PDI [-]
**DTP-P**	21,900	41,700	1.90
**DTP-2T**	16,000	44,900	2.81

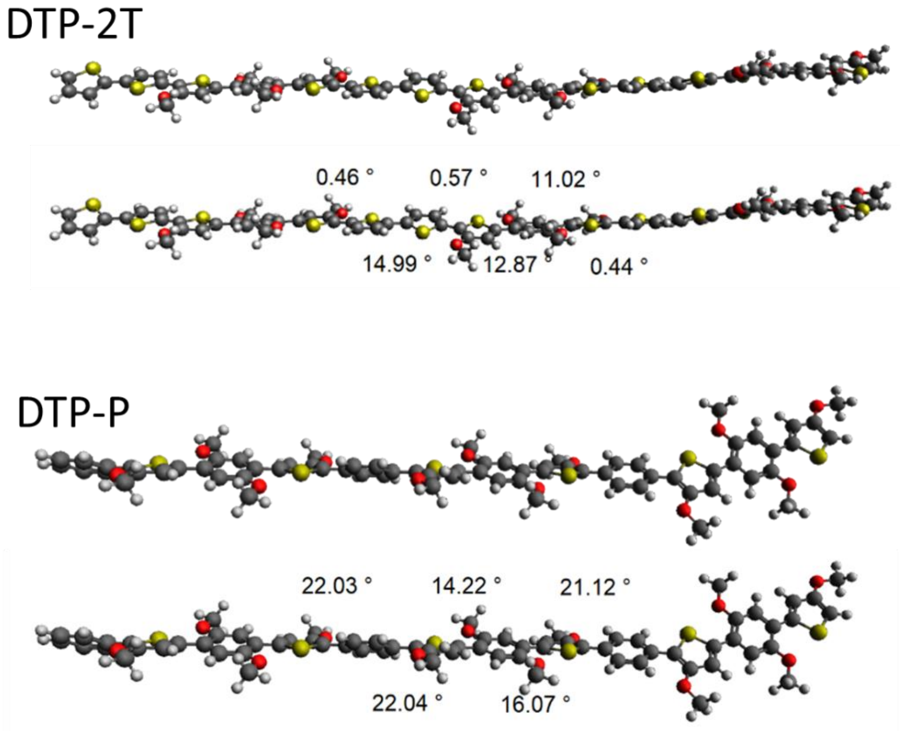

Supplementary Figure 1. Dihedral angles across the polymer backbone as obtained from computational simulations.

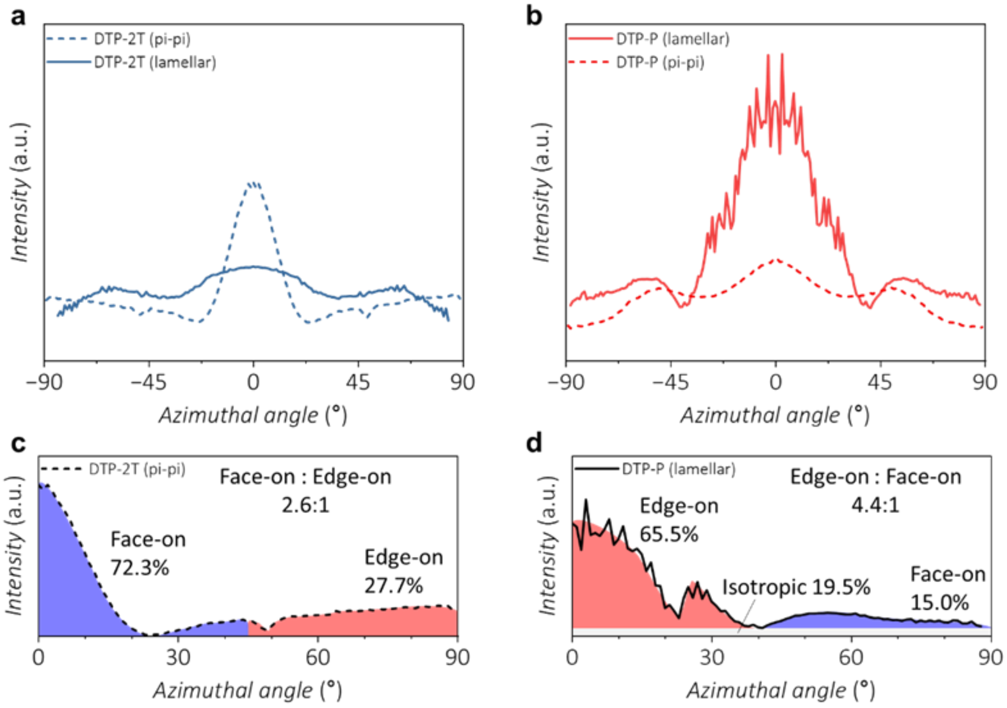

Supplementary Figure 2. Pole figures of lamellar (100) and π–π stacking (010) peaks for **a** DTP-2T and **b** DTP-P. Quantitative analysis of the population ratio of edge-on, face-on, and isotropic crystals for **c** DTP-2T (π–π stacking (010) peak) and **d** DTP-P (lamellar-stacking (100) peak).

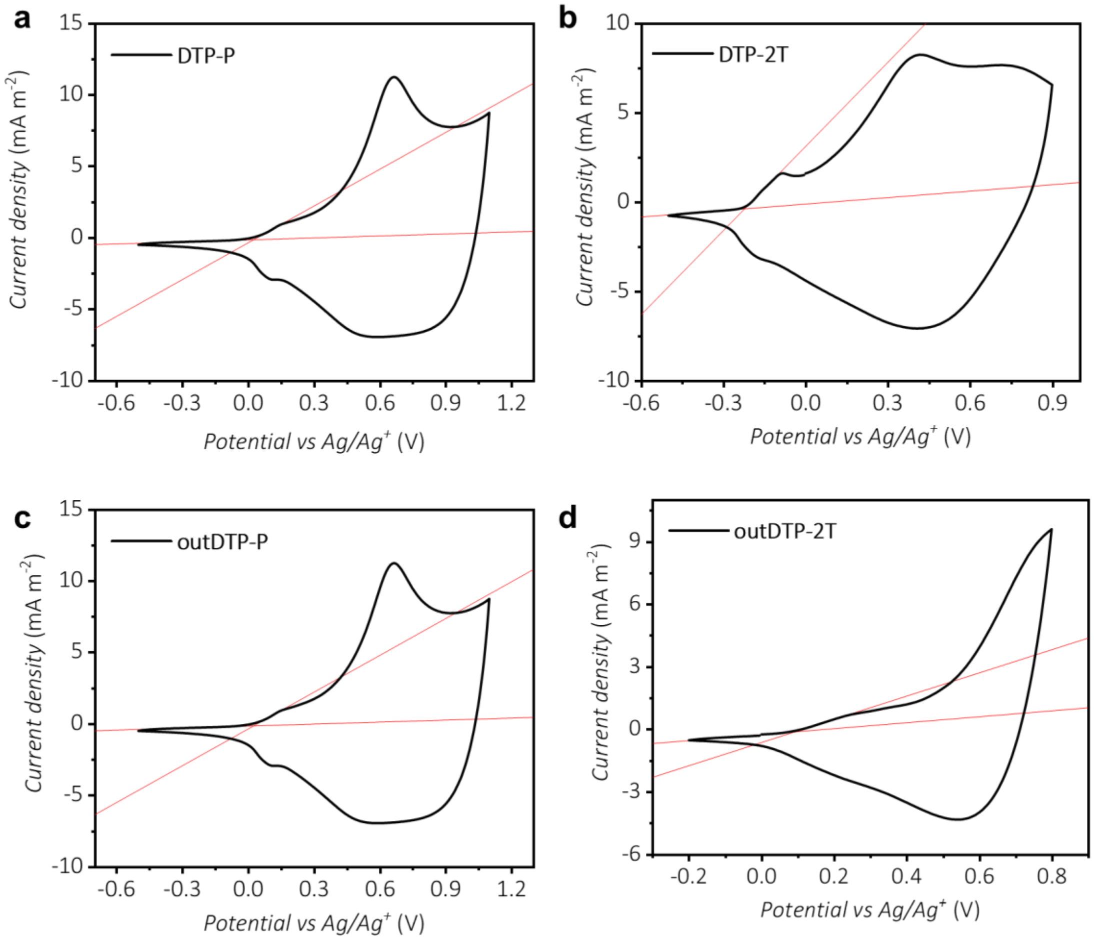

Supplementary Figure 3. Cyclic voltammograms of polymer films prepared by using chloroform solution (5 mg mL^{-1}) with a scan rate of 50 mV s^{-1} versus Ag/Ag^+ reference electrode (a, b) with 0.1 M $TBAPF_6$ as electrolyte in acetonitrile and (c, d) with 0.1 NaCl as electrolyte in water. Note that the red-colored tangents are used to determine the onsets of oxidation.

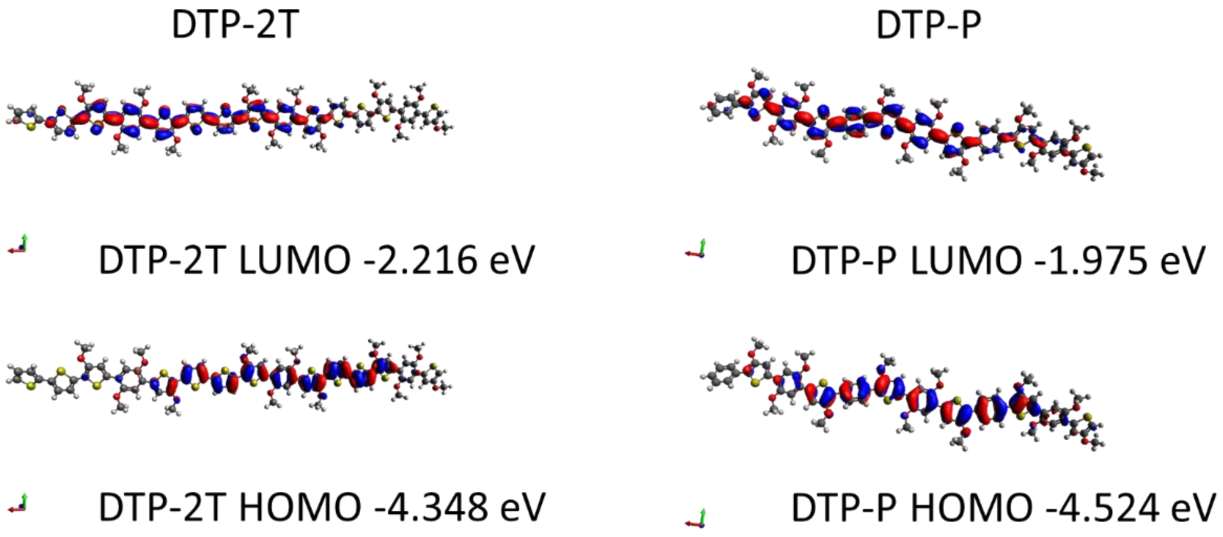

Supplementary Figure 4. Spatial distributions of the HOMO and LUMO of the polymers.

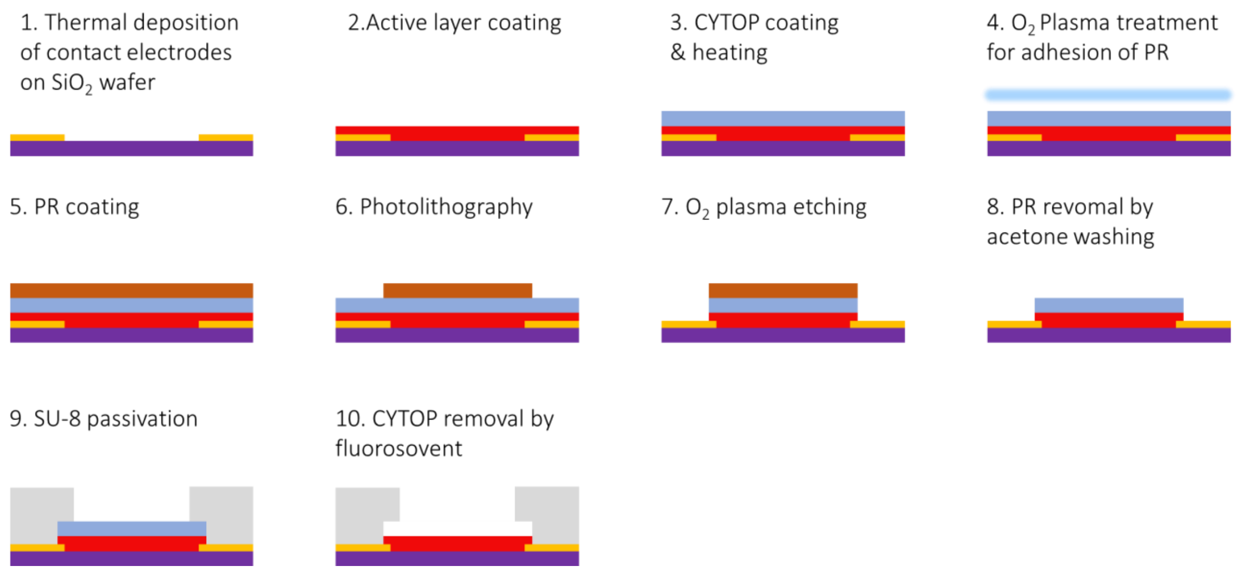

Supplementary Figure 5. Fabrication procedure of organic electrochemical transistor through orthogonal patterning process using CYTOP.

Organic Electrochemical Transistor Fabrication and Characterization Device fabrication procedure is as follows. After sequential ultrasonic cleaning of p-Si^{++}/SiO_2 (300 nm) substrates by placing them in a bath of acetone and isopropanol for 5 min each, source and drain contact electrodes [thermally evaporated Cr (5 nm)/Au (40 nm)] were defined via conventional photolithography. Active polymer films were fabricated by spin-casting on the substrate with patterned contact electrode. To protect the polymer film during patterning process, CYTOP thin film was fabricated on the active polymer layer by spin-casting. Prior to the photolithography process, O_2 plasma treatment (5 sccm, 100 W, 10 s) was conducted to improve the adhesion of photoresist to the CYTOP layer. The positive photoresist (GXR-601, Microchemical GmbH) pattern was prepared on the active polymer layer, and the non-channel area was removed by dry-etching. After the residual photoresist removal, SU-8 photoresist (Microchemicals GmbH) was used to passivate the outer side of electrode from the electrolyte. Finally, CYTOP layer is removed by dipping the devices into fluorosolvent (Novec 7300, 3M) for 3 h with stirring. The channel width was 80 μm defined by positive photoresist pattern, and the length of channel was varied from 20 μm to 80 μm by the contact electrode pattern. Note that the effective capacitance of electrical double layer at the interface between metal electrodes and aqueous electrolyte is negligible compared with the effective capacitance of polymer channel. All devices were measured under N_2 condition using two Keithley 2400 source meters controlled by Matlab software, while

an Ag/AgCl electrode and the solution of 0.1 M NaCl were employed as non-polarizable gate electrode and electrolyte, respectively.

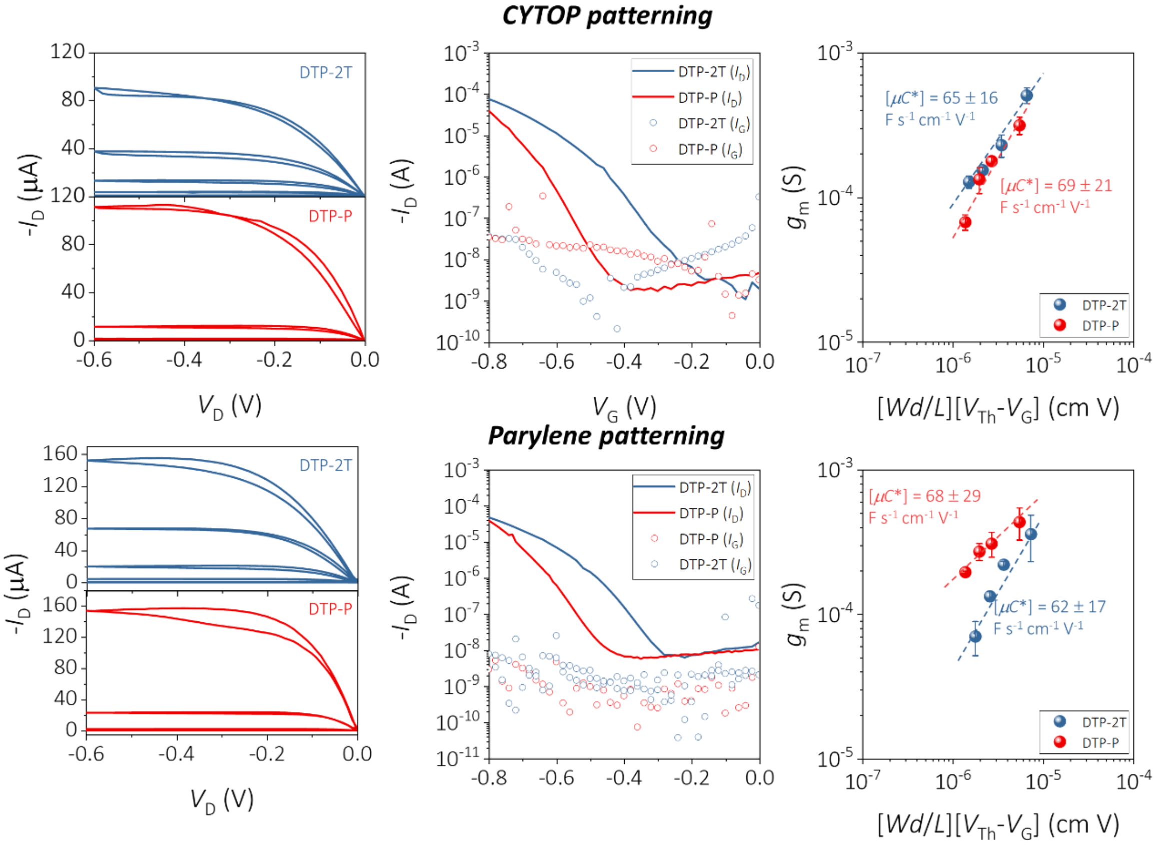

Supplementary Figure 6. Comparison of charge transport characteristics of organic electrochemical transistors fabricated with CYTOP and parylene patterning method.

Organic Electrochemical Transistor Parameterization The thickness of active layer was measured by stylus profiler (DektakXT, Bruker) using at least five samples. The peak transconductance (g_m) values were obtained at a gate voltage = –0.8 V because the aqueous electrolyte limits the voltage window to below –0.8 V due to the electrolysis of water. Furthermore, the peak transconductance values of both polymers were taken at –0.8 V after confirming that the extracted μC^* (which is proportional to g_m) at –0.8 V were in the saturated plateau region (Figure S6). Note that the peak transconductance values were extracted from at least five devices with the channel length of 20 μm. The average μC^* values were obtained from the slope in the scattered plot of transconductance vs. $(Wd/L)(V_{Th}–V_G)$ by linear fitting (4 devices included). The on/off current ratios were obtained from at least five transfer curves measured from OECT devices with the channel length of 20 μm. The volumetric capacitances were calculated using the Bode plots

which were obtained from EIS measurements. The capacitance value of each polymer was measured at different V_{offset}, using at least five samples. The OECT mobility (Figure 2f) was calculated by dividing the average μC^* values with the average C^* values. The threshold voltage values were also extracted from at least five OECT devices with different channel length (20-80 μm).

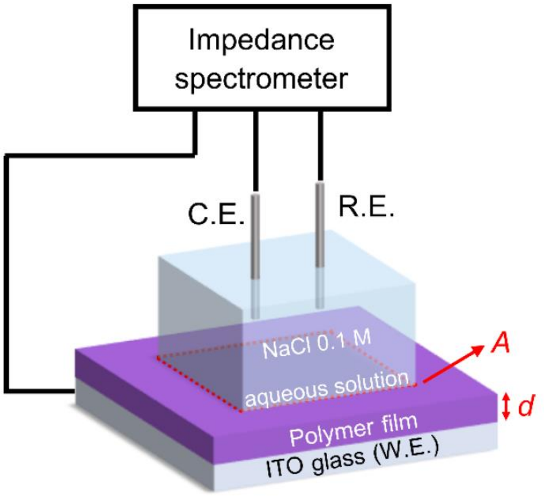

Supplementary Figure 7. Schematic illustration of electrochemical impedance spectroscopy (EIS) measurement. The nominal volume of the polymer film is obtained from the film thickness (*d*) measured by surface profiler and the area (*A*) exposed to the aqueous electrolyte solution during the electrochemical impedance spectroscopy measurement.

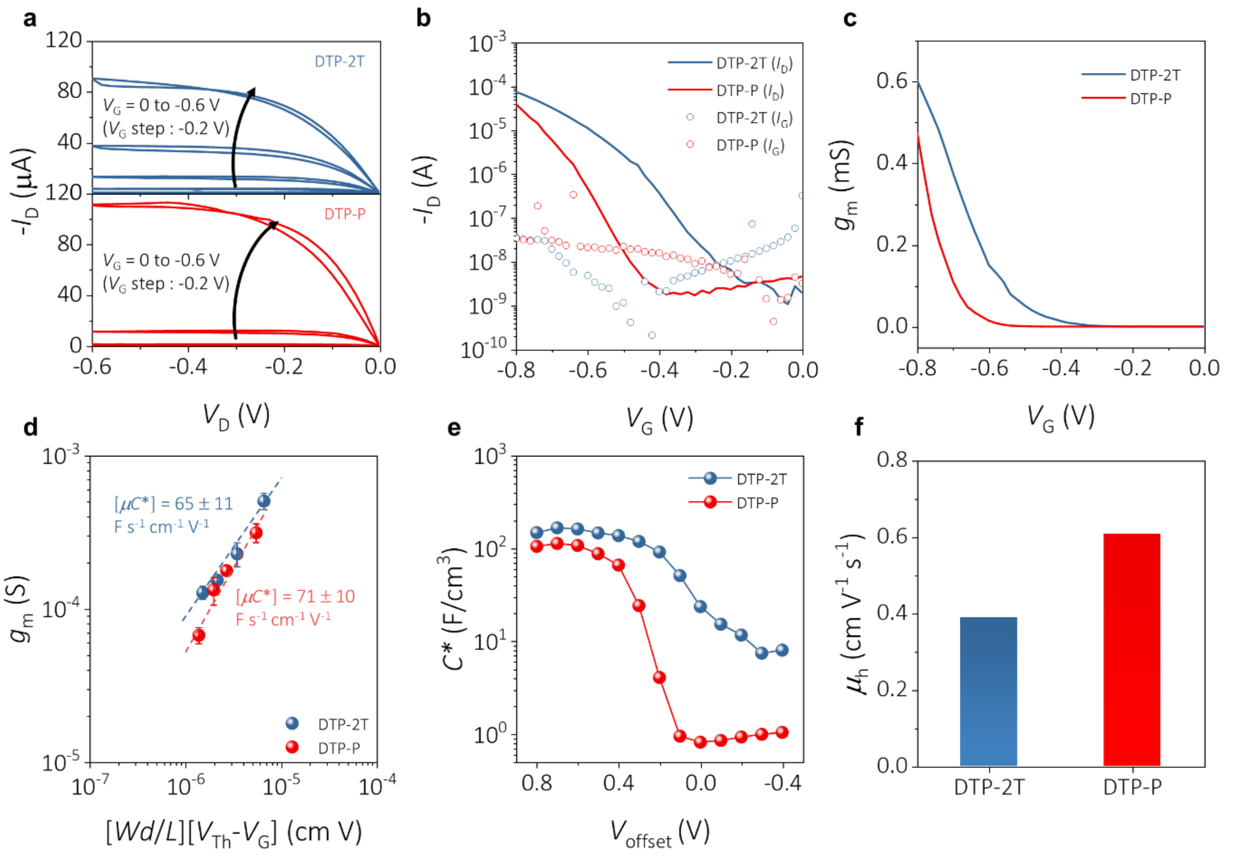

Supplementary Figure 8. Electrical characterization of DTP-based OECTs. a) Representative output curves of the DTP-2T and DTP-P OECTs when VG was scanned from 0 to –0.6 V (Vg step : –0.2 V). Representative b) transfer and c) corresponding transconductance curves of the DTP-2T-and DTP-P-based devices. d) Plot of transconductance versus channel geometry and operating voltage conditions for DTP-2T and DTP-P from which their corresponding μC* were obtained. e) as a function of bias (Voffset of working electrode for C*). f) OECT hole mobility calculated by dividing μC* obtained from OECT characterization by C* extracted from EIS measurement.

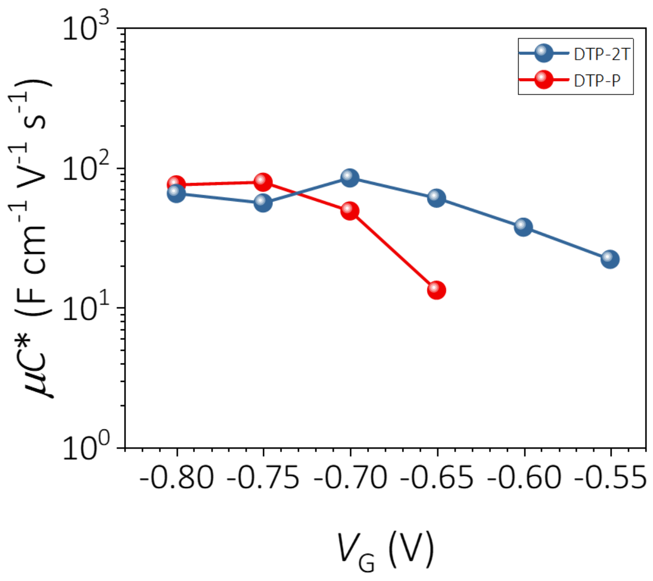

Supplementary Figure 9. Plot of μC^* as a function of gate voltage. At the condition of V_G = –0.8 V, μC^* is in saturation.

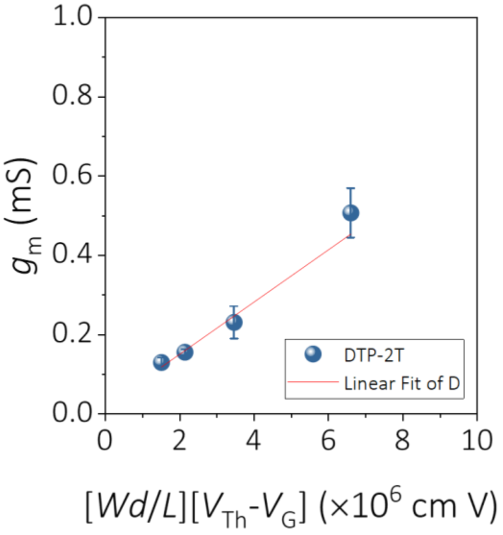

Equation	y = a + b*x
Plot	D
Weight	Instrumental
Intercept	1.58085E-5 ± 2.37
Slope	65.92053 ± 11.075
Residual Sum of Sq	2.12257
Pearson's r	0.97291
R-Square (COD)	0.94656
Adj. R-Square	0.91984

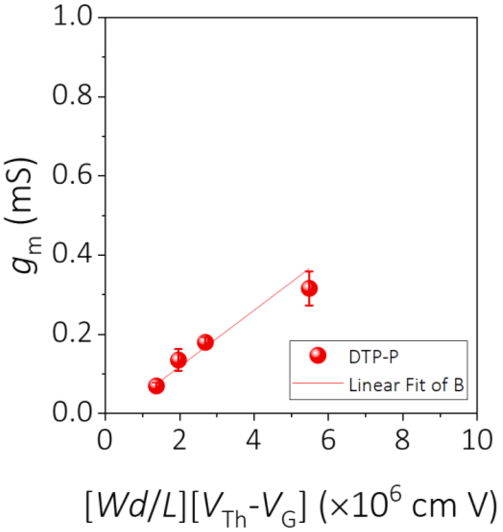

Equation	y = a + b*x
Plot	B
Weight	Instrumental
Intercept	-2.63214E-5 ± 2.052
Slope	71.00532 ± 10.1255
Residual Sum of Sq	3.03765
Pearson's r	0.98026
R-Square (COD)	0.96092
Adj. R-Square	0.94138

Supplementary Figure 10. Linear plots of transconductance versus channel geometry and operating voltage condition for DTP-P and DTP-2T from which their corresponding μC* were obtained. Linear fitting curve and parameters are summarized in the adjacent table.

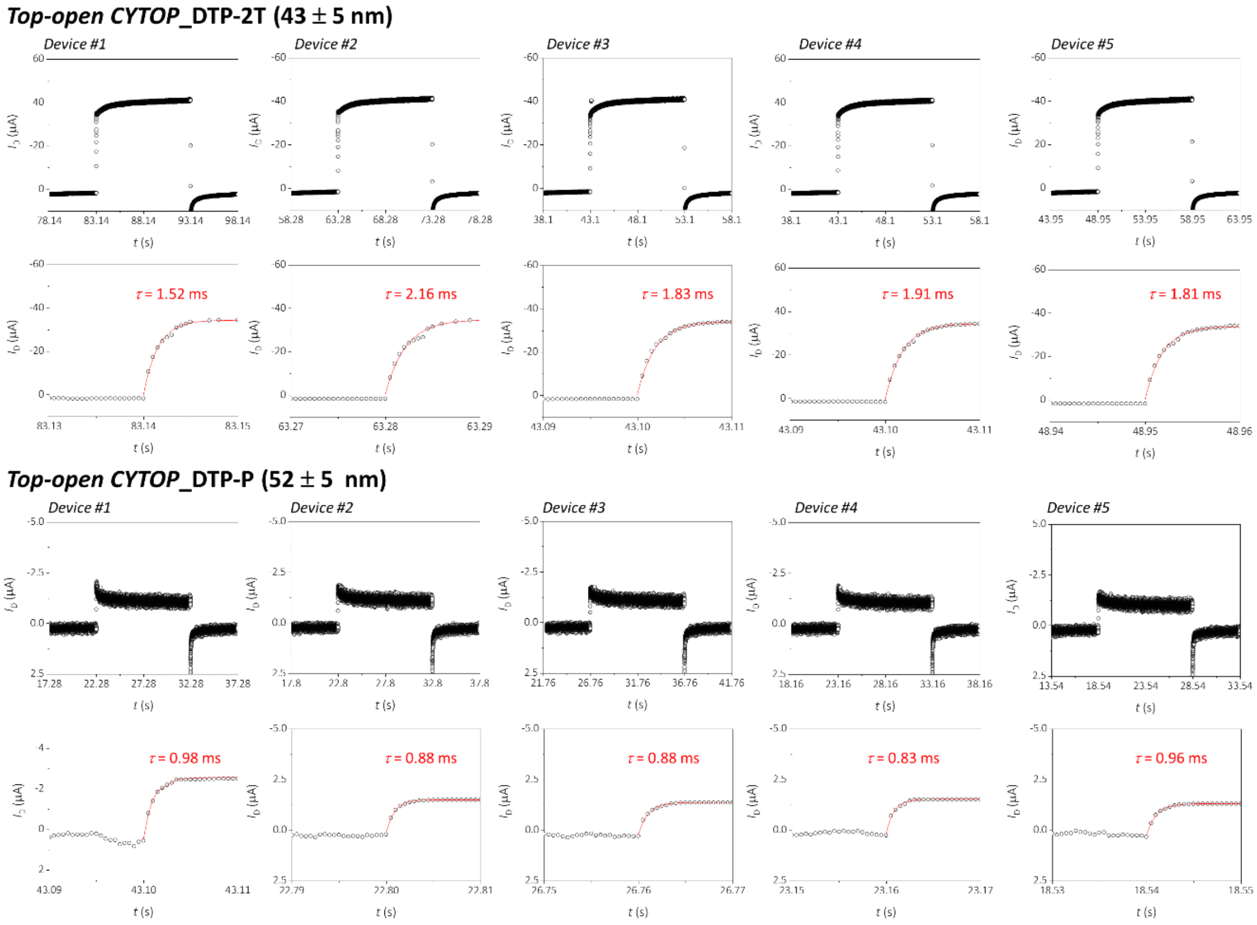

Supplementary Figure 11. Determination of rise time constants from the transient responses of DTP-2T and DTP-P OECT devices fabricated by the orthogonal patterning method. Transient drain current at V_D = –0.6 V was recorded with the V_G = –0.8 V pulse applied. Each red line corresponds to the exponential fitting result for rise time extraction. Upper panels show the full range of drain current responses while lower panels show the magnified view of drain current responses with the corresponding fitting results.

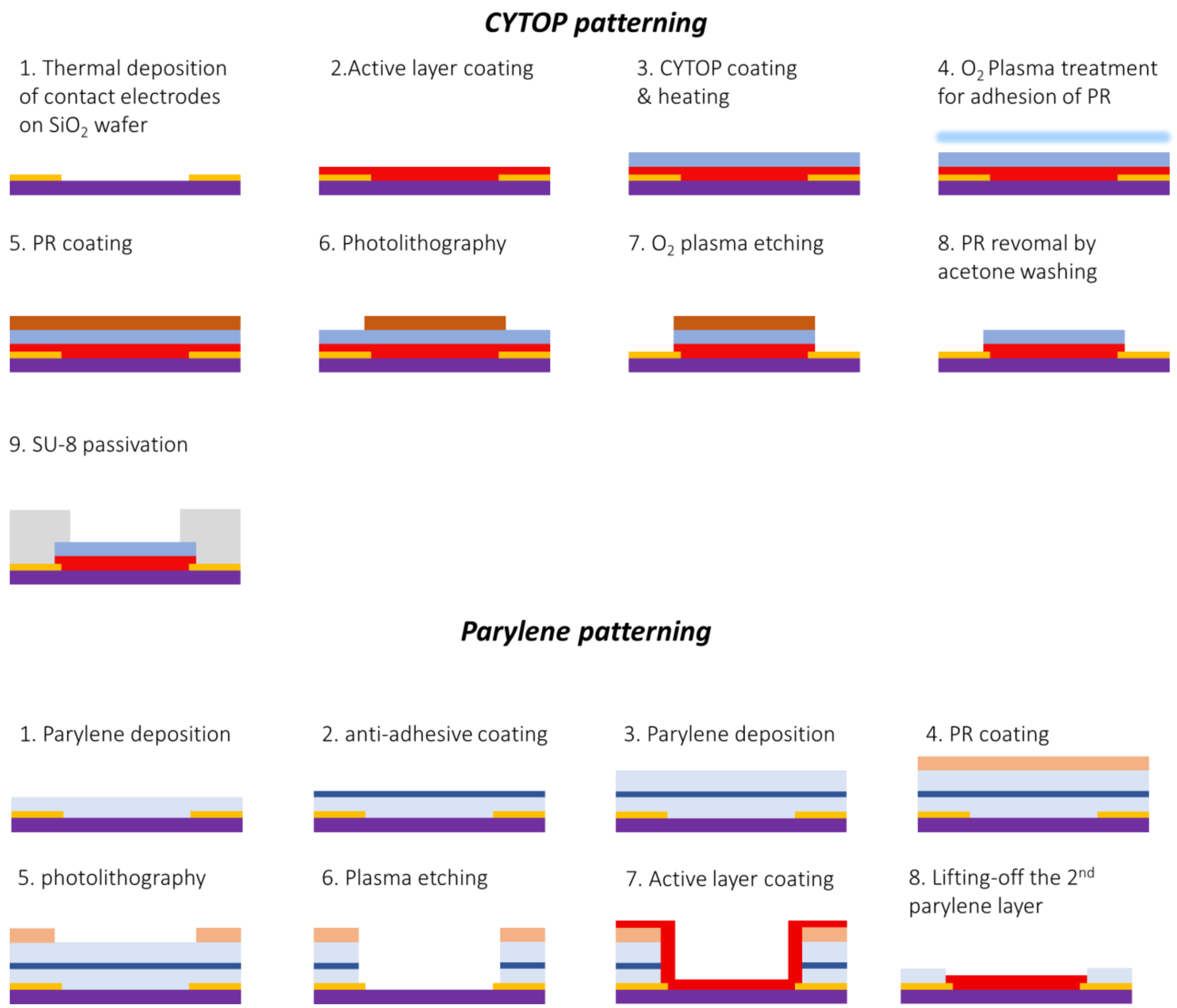

Supplementary Figure 12. Comparison of CYTOP patterning method and parylene patterning method.

Device Fabrication In order to investigate the molecular orientation dependency, we fabricated the device where particular direction of ion injection is blocked by passivation layer as follows. i) side-open devices: All procedure is identical to the device fabrication procedure above except for CYTOP removal step. By leaving the CYTOP layer on the top of active polymer layer, side-open device fabrication is completed. ii) top-open devices: After p-Si^{++}/SiO_2 (300 nm) cleaning process by sequential ultrasonication with acetone and isopropanol, source and drain contact electrodes were prepared by combining thermal evaporation [Cr (5 nm)/Au (40 nm)] and photolithography. HMDS treatment was conducted to the substrate with electrode pattern to improve the adhesion of parylene layer, followed by parylene layer (2 μm) deposition on the substrate by using parylene coating system (LAVIDA-110H, Femto Science, Republic of Korea). Anti-adhesive layer is

fabricated by spin-casting of fluorosolvent, and second parylene layer (2 μm) was also deposited on it. Prior to photolithography, O_2 plasma treatment was conducted to improve the adhesion of photoresist to the parylene layer. Then, positive photoresist (AZ9260, AZ Electronic Materials) pattern was prepared on the parylene film to define the channel, and the channel area was removed by dry-etching. Active polymer layers were fabricated by spin-casting on the patterned parylene layer, followed by the removal of second parylene layer by peeling off.

Measurement of Transient/Frequency Response of Drain Current Transient drain current measurement was conducted by applying V_G pulse of -0.8 V using function generator (AFG 3021B, Tektronix) and measuring I_D at a constant V_D of -0.6 V with source meter (Kiethley2400 Tektronix), low noise current preamplifier (SR570, Stanford Research Systems), and data acquisition system (Axon Digidata 1550B, Molecular Devices). To measure high-frequency signal, the I_D was converted to voltage signal by using low noise current preamplifier, and then the voltage signal is recorded by the data acquisition system. In case of frequency response, measurement was conducted by applying V_G of sine wave with amplitude of 10 mV using function generator. I_D measurement setup is identical to the transient measurement setup. Additionally, all transient/frequency response measurements were conducted under N_2 atmosphere.

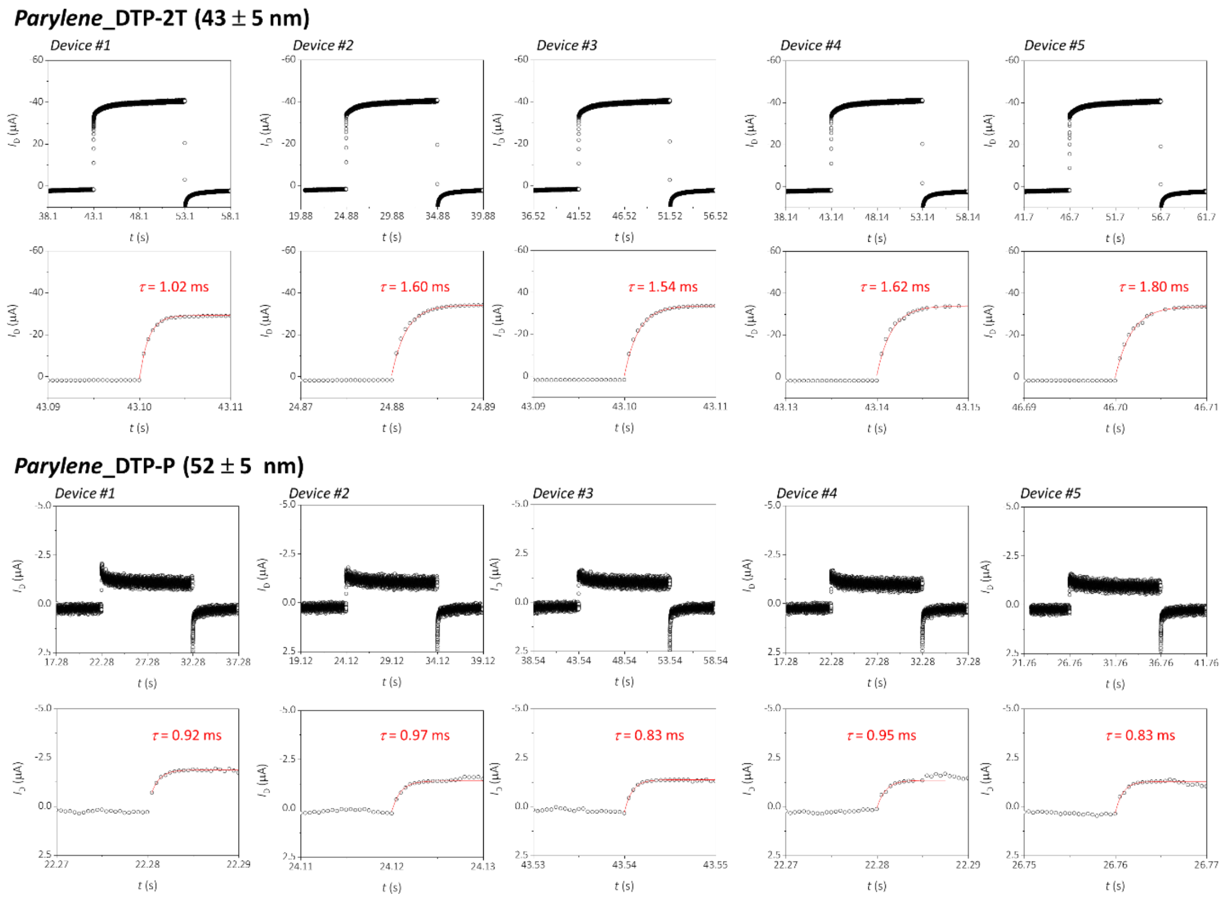

Supplementary Figure 13. Determination of rise time constants from the transient responses of DTP-2T and DTP-P OECT devices fabricated by the parylene patterning method. Transient drain current at V_D = –0.6 V was recorded with the V_G = –0.8 V pulse applied. Each red line is the exponential fitting result to extract the rise time. Upper panel shows the full range of drain current response from each OECT device.

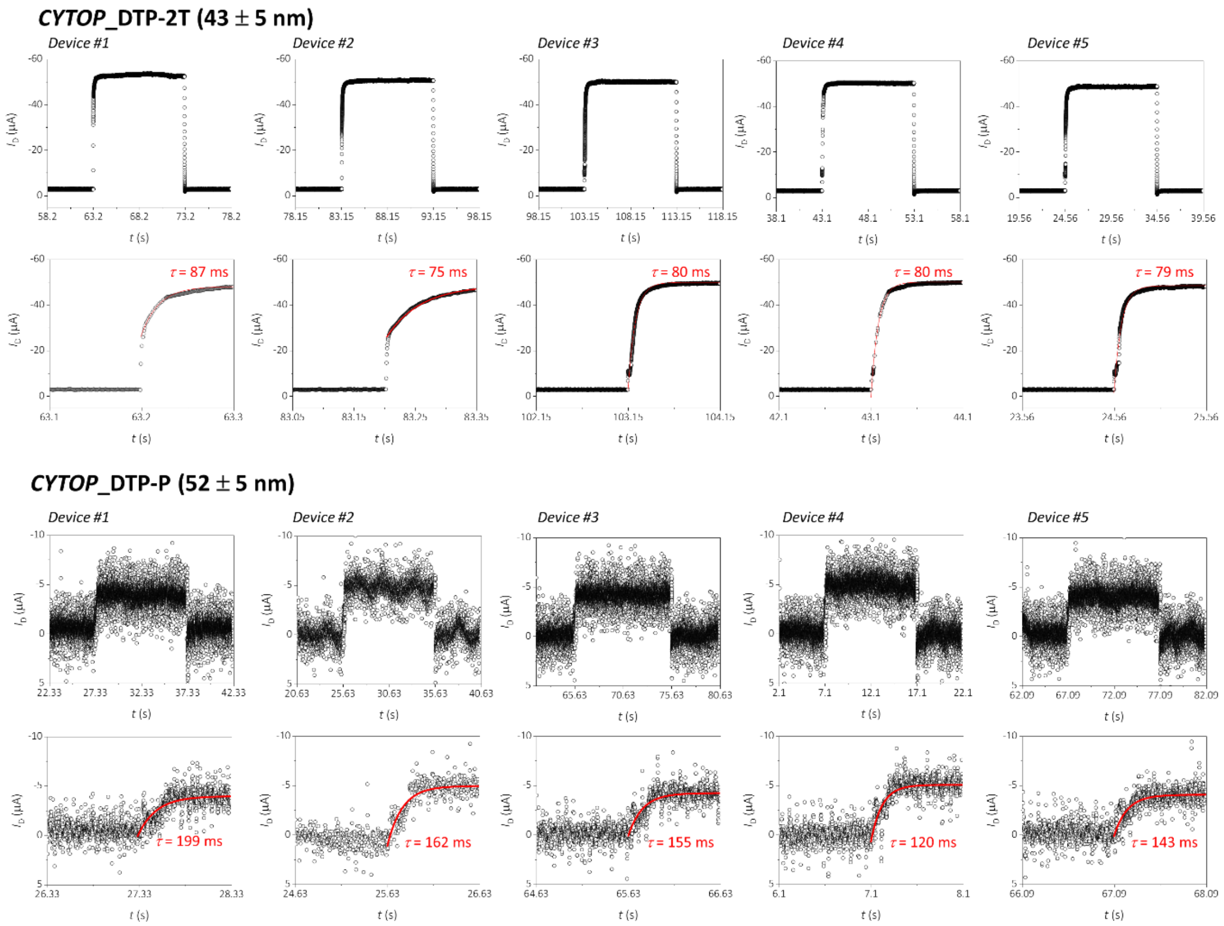

Supplementary Figure 14. Determination of rise time constants from the transient responses of DTP-2T and DTP-P OECT devices fabricated by the CYTOP patterning method. Transient drain current at V_D = –0.6 V was recorded with the V_G = –0.8 V pulse applied. The red line denotes an exponential fitting to extract the corresponding rise time. Upper panel shows the full range of drain current response from each OECT device.

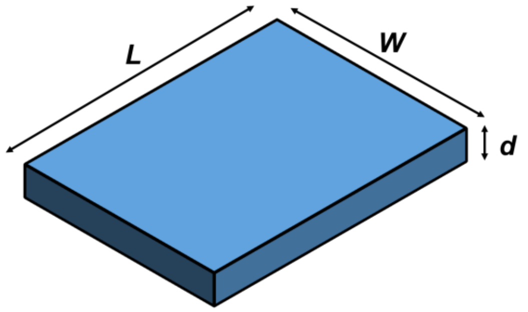

Time constant is proportional to
[(*depth of ion penetration*)*(surface area)^(1/2)].

Parylene Devices
(side wall blocked)

- *depth of ion penetration = d*
- *surface area = WL*

CYTOP Devices
(top-surface blocked)

- *depth of ion penetration* = (*WL*)/2(*W*+*L*)

Assuming ion penetration occurs from the side wall to the center of the active layer as shown in below,

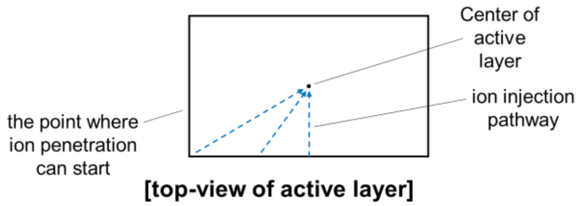

[top-view of active layer]

the depth of ion penetration is depending on the point where ion penetration can start.

Therefore, the average depth of ion penetration can be obtained by dividing the sum of the distances from any point where ion penetration can start to the center of the active layer by the number of arbitrary points where ion penetration can start.

The sum of the distances from any point where ion penetration can start to the center of the active layer is equal to the top-surface area of active layer, which is *WL* and the number of arbitrary points where ion penetration can start is equal to the rectangle's perimeter, which is equal to 2(*W*+*L*).

Therefore, the average depth of ion penetration is equal to (*WL*)/2(*W*+*L*)

- *surface area = 2(dL + dW)*

Supplementary Figure 15. A schematic representation of active layer and the derivation of the average depth of ion penetration.

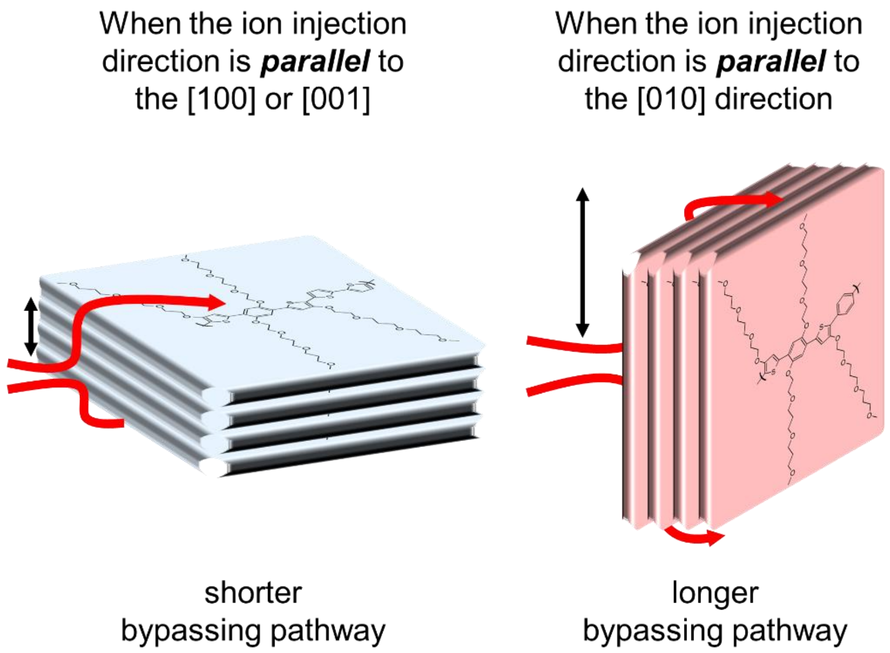

Supplementary Figure 16. A schematic representation of ion bypassing pathway regarding ion injection direction.

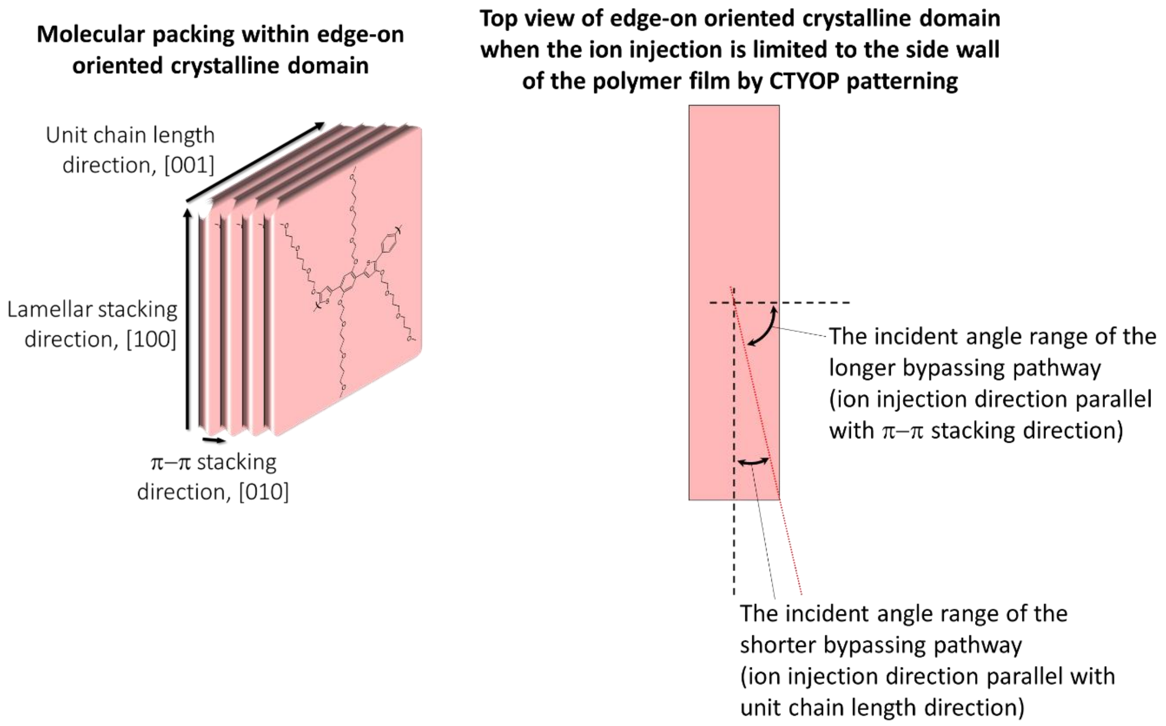

Supplementary Figure 17. Schematic illustration of molecular packing within edge-on oriented crystalline domain. The diagram also shows the incident angle range when the ion injection occurs in the DTP-P device patterned with CYTOP.

Supplementary Table 1. Rise time constants and other fitting parameters extracted from OECT devices fabricated by the orthogonal patterning method (Figure S11).

Polymer	Parameters	Device #1	Device #2	Device #3	Device #4	Device #5
DTP-2T	$I_{D,0}$	-0.686	-0.695	-0.682	-0.688	-0.677
	A	0.697	0.691	0.673	0.700	0.679
	τ	1.52e-3	2.16e-3	1.81e-3	1.83e-3	1.81e-3
DTP-P	$I_{D,0}$	-0.025	-0.029	-0.027	-0.030	-0.026
	A	0.030	0.032	0.031	0.034	0.030
	τ	9.8e-4	8.8e-4	9.2e-4	8.3e-4	9.6e-4

Supplementary Table 2. Rise time constants and other fitting parameters extracted from OECT devices fabricated by the parylene patterning method.

Polymer	Parameters	Device #1	Device #2	Device #3	Device #4	Device #5
DTP-2T	$I_{D,0}$	-0.584	-0.682	-0.671	-0.678	-0.674
	A	0.612	0.670	0.674	0.663	0.680
	τ	1.02e-3	1.60e-3	1.54e-3	1.62e-3	1.80e-3
DTP-P	$I_{D,0}$	-0.037	-0.027	-0.027	-0.026	-0.025
	A	0.039	0.030	0.033	0.028	0.032
	τ	9.2e-4	9.7e-4	8.3e-4	9.5e-4	8.3e-4

Supplementary Table 3. Rise time constants and other fitting parameters extracted from OECT devices fabricated by the CYTOP patterning method.

Polymer	Parameters	Device #1	Device #2	Device #3	Device #4	Device #5
DTP-2T	$I_{D,0}$	-1.04	-1.00	-0.99	-1.00	-0.96
	A	0.927	0.935	0.961	1.016	0.948
	τ	0.087	0.075	0.080	0.080	0.079
DTP-P	$I_{D,0}$	-0.791	-0.989	-0.845	-1.013	-0.812
	A	0.745	0.716	0.866	0.755	0.842
	τ	0.199	0.162	0.155	0.120	0.143

Supplementary Table 4. Solid-state packing parameters extracted from the GIWAXD measurements.

Polymer	Lamellar stacking (100)			π−π stacking (010)			Unit chain length (001)		
	q [Å^{-1}]	d [Å]	L_c [Å]	q [Å^{-1}]	d [Å]	L_c [Å]	q [Å^{-1}]	d [Å]	L_c [Å]
DTP-2T	0.323	19.5	76.1	1.711	3.7	33.8			
DTP-P	0.373	17.1	62.6	1.439	4.4	10.9	0.412	15.3	86.7

Supplementary Movie 1. (separate file)

Movie clip recorded during the moving front experiment of DTP-P.

Supplementary Movie 2. (separate file)

Movie clip recorded during the moving front experiment of DTP-2T.

Supplementary References

1. Paquin, F., Rivnay, J., Salleo, A., Singerliin, N., Silva, C. Multi-phase microstructures drive exciton dissociation in neat semicrystalline polymeric semiconductors. *J. Mater. Chem. C* **3**, 10715-10722 (2015).
2. Sheik, D. A., Brooks, L., Frantzen, K., Dewhurst, S., Yang, J. Inhibition of the enhancement of infection of human immunodeficiency virus by semen-derived enhancer of virus infection amyloid-targeting polymeric nanoparticles. *ACS Nano* **9**, 1829-1836 (2015).
