# Supplementary material for: Peculiar transient behaviors of organic electrochemical transistors governed by ion injection directionality
Source: Nat Commun. 2023 Nov 28;14:7577. doi: 10.1038/s41467-023-42840-z (PMC10684893; doi:10.1038/s41467-023-42840-z)
Supplement: Supplementary file 4 — Description of Additional Supplementary Files [file 41467_2023_42840_MOESM4_ESM.pdf]

Description of Additional Supplementary Files

Supplementary Movie 1: Movie clip recorded during the moving front experiment of DTP-P.

Supplementary Movie 2: Movie clip recorded during the moving front experiment of DTP-2T
